# Supplementary figures and images for: Direct cost of systemic arterial hypertension and its complications in the circulatory system from the perspective of the Brazilian public health system in 2019
Source: PLoS One. 2021 Jun 10;16(6):e0253063. doi: 10.1371/journal.pone.0253063 (PMC8191920; doi:10.1371/journal.pone.0253063)

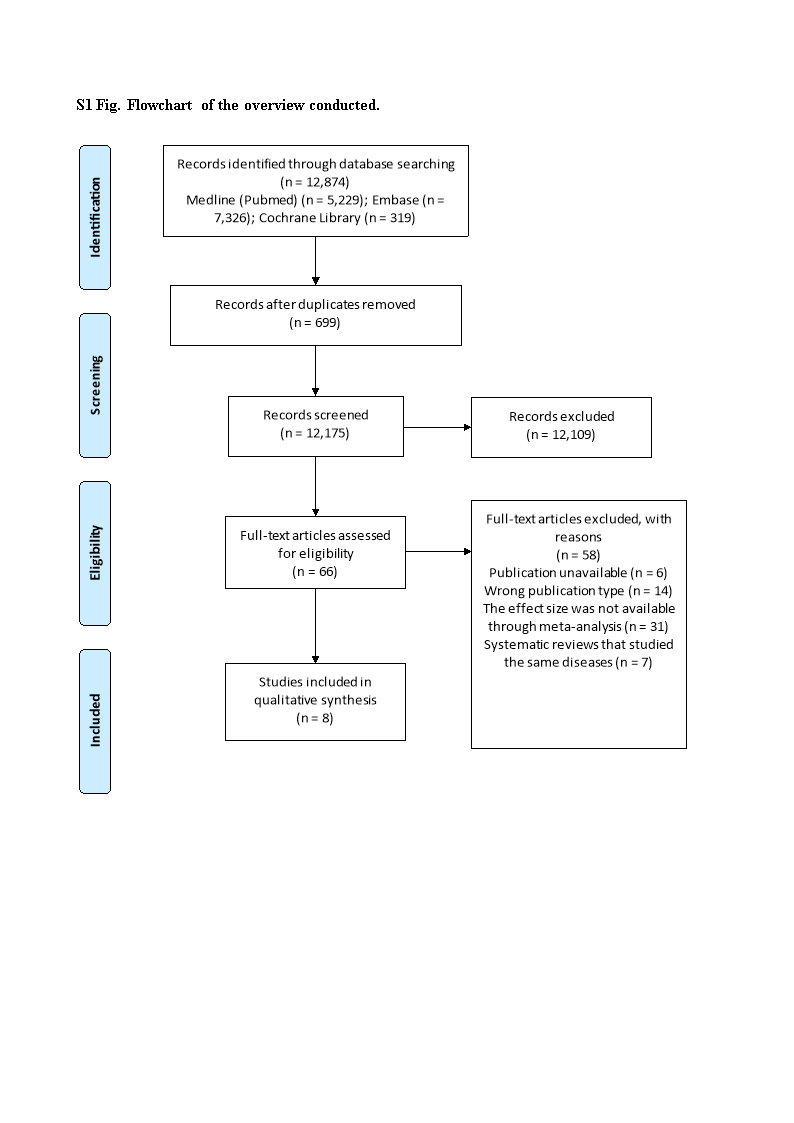

Supplement: S1 Fig — (TIF) [file pone.0253063.s001.tif]
