# Supplementary material for: Direct cost of systemic arterial hypertension and its complications in the circulatory system from the perspective of the Brazilian public health system in 2019
Source: PLoS One. 2021 Jun 10;16(6):e0253063. doi: 10.1371/journal.pone.0253063 (PMC8191920; doi:10.1371/journal.pone.0253063)
Supplement: S1 Table — Brazil, 2019. (DOCX) [file pone.0253063.s002.docx]

**S1 Table. Estimated hypertensive population using antihypertensive drugs in SUS primary care. Brazil, 2019.**

|  | Parameter | Population | Source |
| --- | --- | --- | --- |
| Number of hypertensive patients in primary care |  |  |  |
| Brazilian population ≥20 years old |  | 149,717,521 | IBGE |
| Prevalence of systemic arterial hypertension in Brazil | 0.2370 | 35,483,052 | PNAUM |
| Hypertensive patients with antihypertensive drugs prescription | 0.9370 | 33,247,620 | PNAUM |
| Hypertensive patients who did not intentionally abandon treatment with antihypertensive drugs | 0.8940 | 29,723,372 | PNAUM |
| Hypertensive patients using antihypertensive drugs | 0.9460 | 28,118,310 | PNAUM |
| Sources of antihypertensive drugs |  |  |  |
| Public pharmacies | 0.5600 | 15,746,254 | PNAUM |
| Brazilian Popular Pharmacy Program (PFPB) | 0.1830 | 5,145,651 | PNAUM |
| Private pharmacies (out-of-pocket) | 0.2570 | 7,226,406 | PNAUM |
| Number of drugs used by hypertensive patients obtained at public pharmacies |  |  |  |
| One | 0.5469 | 8,611,626 | PNAUM |
| Twos | 0.3740 | 5,889,099 | PNAUM |
| Three | 0.0791 | 1,245,529 | PNAUM |
| Number of drugs used by hypertensive patients obtained in the PFPB |  |  |  |
| One | 0.5469 | 2,814,157 | PNAUM |
| Two | 0.3740 | 1,924,473 | PNAUM |
| Three | 0.0791 | 407,021 | PNAUM |
