# Supplementary material for: Direct cost of systemic arterial hypertension and its complications in the circulatory system from the perspective of the Brazilian public health system in 2019
Source: PLoS One. 2021 Jun 10;16(6):e0253063. doi: 10.1371/journal.pone.0253063 (PMC8191920; doi:10.1371/journal.pone.0253063)
Supplement: S2 Table — Brazil, 2019. (DOCX) [file pone.0253063.s003.docx]

**S2 Table. Estimated costs of antihypertensive drugs from public pharmacies in primary care. Brazil, 2019.**

| Drug | Concentration per unit (mg) | DDD (mg) | Units per day | Units per year | Base case (Int$) | Lower limit (Int$) | Upper limit (Int$) |
| --- | --- | --- | --- | --- | --- | --- | --- |
| Amlodipine | 5 | 5 | 1 | 365 | 0.01195 | 0.01136 | 0.01818 |
| Atenolol | 50 | 75 | 2 | 730 | 0.01691 | 0.01678 | 0.02273 |
| Captopril | 25 | 50 | 2 | 730 | 0.01018 | 0.00909 | 0.01364 |
| Enalapril | 10 | 10 | 1 | 365 | 0.01409 | 0.01364 | 0.02591 |
| Furosemide | 40 | 40 | 1 | 365 | 0.01436 | 0.01455 | 0.02273 |
| Hydrochlorothiazide | 25 | 25 | 1 | 365 | 0.00605 | 0.00682 | 0.01364 |
| Losartan | 50 | 50 | 1 | 365 | 0.02627 | 0.02273 | 0.03636 |
| Nifedipine | 10 | 10 | 1 | 365 | 0.01555 | 0.01455 | 0.05114 |
| Propranolol | 40 | 160 | 4 | 1460 | 0.01377 | 0.01136 | 0.01818 |

**Source:** Brazil. Ministry of Health. Health Prices Database – BPS, 2020. Note: mg - milligrams; DDD - Defined daily dose.
