# Supplementary material for: Direct cost of systemic arterial hypertension and its complications in the circulatory system from the perspective of the Brazilian public health system in 2019
Source: PLoS One. 2021 Jun 10;16(6):e0253063. doi: 10.1371/journal.pone.0253063 (PMC8191920; doi:10.1371/journal.pone.0253063)
Supplement: S3 Table — Brazil, 2019. (DOCX) [file pone.0253063.s004.docx]

**S3 Table. Estimated costs of antihypertensive drugs from the Brazilian Popular Pharmacy Program (PFPB) in primary care. Brazil, 2019.**

| Drug | Concentration per unit (mg) | DDD (mg) | Units per day | Units per year | Base case (Int$) | Lower limit (Int$) | Upper limit (Int$) |
| --- | --- | --- | --- | --- | --- | --- | --- |
| Atenolol | 25 | 75 | 3 | 1095 | 0.04545 | 0.03636 | 0.05455 |
| Captopril | 25 | 50 | 2 | 730 | 0.04545 | 0.03636 | 0.05909 |
| Enalapril | 10 | 10 | 1 | 365 | 0.07727 | 0.05909 | 0.09091 |
| Hydrochlorothiazide | 25 | 25 | 1 | 365 | 0.02727 | 0.02273 | 0.02727 |
| Losartan | 50 | 50 | 1 | 365 | 0.07727 | 0.06364 | 0.09545 |
| Propranolol | 40 | 160 | 4 | 1460 | 0.03182 | 0.02727 | 0.03636 |

**Source:** Brazil. Ministry of Health. Ordinance No. 739, of March 27, 2018, which updates the reference values of drugs provided by the Brazilian Popular Pharmacy Program Here to treat hypertension, diabetes mellitus and asthma. Note: mg - milligrams; DDD - Defined daily dose.
