# Supplementary material for: Direct cost of systemic arterial hypertension and its complications in the circulatory system from the perspective of the Brazilian public health system in 2019
Source: PLoS One. 2021 Jun 10;16(6):e0253063. doi: 10.1371/journal.pone.0253063 (PMC8191920; doi:10.1371/journal.pone.0253063)
Supplement: S4 Table — Brazil, 2019. (DOCX) [file pone.0253063.s005.docx]

**S4 Table. Distribution of use and estimated total cost of antihypertensive drugs in primary care. Brazil, 2019.**

|  |  |  | Public pharmacies | |  | Brazilian Popular Pharmacy Program (PFPB) | | | | |
| --- | --- | --- | --- | --- | --- | --- | --- | --- | --- | --- |
| **One drug** | **Base case** | **Lower limit** | **Upper limit** | **Population** | **Total cost (Int$)** | **Base case** | **Lower limit** | **Upper limit** | **Population** | **Total cost (Int$)** |
| Amlodipine | 0.03464 | 0.02400 | 0.04977 | 298343 | 1,301,792.56 | 0.03464 | 0.02400 | 0.04977 | 97494 | 425,406.21 |
| Atenolol | 0.09128 | 0.07661 | 0.10843 | 786071 | 9,702,974.58 | 0.09128 | 0.07661 | 0.10843 | 256877 | 12,785,468.86 |
| Captopril | 0.21966 | 0.19648 | 0.24474 | 1891603 | 14,059,769.21 | 0.21966 | 0.19648 | 0.24474 | 618149 | 20,511,307.73 |
| Enalapril | 0.17746 | 0.15367 | 0.20404 | 1528183 | 7,859,723.02 | 0.17746 | 0.15367 | 0.20404 | 499389 | 14,085,039.75 |
| Furosemide | 0.01344 | 0.00877 | 0.02056 | 115767 | 606,934.81 | 0.01344 | 0.00877 | 0.02056 | 37831 | 198,337.62 |
| Hydrochlorothiazide | 0.12931 | 0.10990 | 0.15157 | 1113609 | 2,457,279.50 | 0.12931 | 0.10990 | 0.15157 | 363912 | 3,622,578.55 |
| Losartan | 0.27722 | 0.25321 | 0.30259 | 2387349 | 22,893,591.75 | 0.27722 | 0.25321 | 0.30259 | 780152 | 22,003,832.55 |
| Nifedipine | 0.01251 | 0.00851 | 0.01836 | 107741 | 611,332.23 | 0.01251 | 0.00851 | 0.01836 | 35208 | 199,773.39 |
| Propranolol | 0.04447 | 0.03649 | 0.05410 | 382959 | 7,700,609.20 | 0.04447 | 0.03649 | 0.05410 | 125146 | 5,813,600.55 |
| **Two drugs** |  |  |  |  |  |  |  |  |  |  |
| Amlodipine \| Atenolol | 0.01318 | 0.00752 | 0.02300 | 77635 | 1,297,051.47 | 0.01318 | 0.00752 | 0.02300 | 25370 | 1,373,433.78 |
| Amlodipine \| Captopril | 0.00379 | 0.00189 | 0.00761 | 22331 | 263,419.52 | 0.00379 | 0.00189 | 0.00761 | 7298 | 274,005.07 |
| Amlodipine \| Enalapril | 0.01675 | 0.01109 | 0.02522 | 98641 | 937,739.63 | 0.01675 | 0.01109 | 0.02522 | 32234 | 1,049,795.45 |
| Amlodipine \| Furosemide | 0.00080 | 0.00025 | 0.00254 | 4730 | 45,437.03 | 0.00080 | 0.00025 | 0.00254 | 1546 | 14,851.09 |
| Amlodipine \| Hydrochlorothiazide | 0.02013 | 0.01330 | 0.03035 | 118532 | 778,755.24 | 0.02013 | 0.01330 | 0.03035 | 38735 | 554,605.97 |
| Amlodipine \| Losartan | 0.03833 | 0.02956 | 0.04957 | 225734 | 3,149,656.24 | 0.03833 | 0.02956 | 0.04957 | 73767 | 2,402,440.30 |
| Amlodipine \| Propranolol | 0.00171 | 0.00051 | 0.00580 | 10095 | 247,040.71 | 0.00171 | 0.00051 | 0.00580 | 3299 | 167,648.43 |
| Atenolol \| Captopril | 0.01280 | 0.00810 | 0.02018 | 75404 | 1,491,216.92 | 0.01280 | 0.00810 | 0.02018 | 24641 | 2,044,082.95 |
| Atenolol \| Enalapril | 0.02410 | 0.01548 | 0.03734 | 141934 | 2,481,974.05 | 0.02410 | 0.01548 | 0.03734 | 46382 | 3,616,741.86 |
| Atenolol \| Furosemide | 0.00192 | 0.00045 | 0.00811 | 11298 | 198,690.74 | 0.00192 | 0.00045 | 0.00811 | 3692 | 203,117.06 |
| Atenolol \| Hydrochlorothiazide | 0.04531 | 0.03337 | 0.06125 | 266840 | 3,882,582.65 | 0.04531 | 0.03337 | 0.06125 | 87199 | 5,208,158.45 |
| Atenolol \| Losartan | 0.04520 | 0.03409 | 0.05970 | 266194 | 5,838,481.40 | 0.04520 | 0.03409 | 0.05970 | 86988 | 6,783,087.00 |
| Atenolol \| Nifedipine | 0.00430 | 0.00145 | 0.01272 | 25336 | 456,497.14 | 0.00430 | 0.00145 | 0.01272 | 8279 | 459,044.21 |
| Atenolol \| Propranolol | 0.00091 | 0.00021 | 0.00403 | 5383 | 174,688.14 | 0.00091 | 0.00021 | 0.00403 | 1759 | 169,263.77 |
| Captopril \| Enalapril | 0.00224 | 0.00058 | 0.00859 | 13197 | 165,964.27 | 0.00224 | 0.00058 | 0.00859 | 4312 | 264,698.00 |
| Captopril \| Furosemide | 0.01427 | 0.00895 | 0.02269 | 84057 | 1,065,460.68 | 0.01427 | 0.00895 | 0.02269 | 27469 | 1,055,483.84 |
| Captopril \| Hydrochlorothiazide | 0.14950 | 0.12787 | 0.17407 | 880437 | 8,486,812.38 | 0.14950 | 0.12787 | 0.17407 | 287714 | 12,410,935.73 |
| Captopril \| Losartan | 0.01022 | 0.00646 | 0.01613 | 60195 | 1,024,655.71 | 0.01022 | 0.00646 | 0.01613 | 19671 | 1,207,531.16 |
| Captopril \| Nifedipine | 0.00338 | 0.00145 | 0.00785 | 19919 | 261,074.71 | 0.00338 | 0.00145 | 0.00785 | 6509 | 252,913.11 |
| Enalapril \| Furosemide | 0.00745 | 0.00465 | 0.01191 | 43888 | 455,816.78 | 0.00745 | 0.00465 | 0.01191 | 14342 | 479,700.79 |
| Enalapril \| Hydrochlorothiazide | 0.14888 | 0.12617 | 0.17486 | 876769 | 6,444,052.88 | 0.14888 | 0.12617 | 0.17486 | 286516 | 10,933,190.09 |
| Enalapril \| Losartan | 0.00382 | 0.00178 | 0.00818 | 22518 | 331,751.55 | 0.00382 | 0.00178 | 0.00818 | 7359 | 415,114.50 |
| Enalapril \| Nifedipine | 0.00259 | 0.00095 | 0.00703 | 15263 | 165,104.03 | 0.00259 | 0.00095 | 0.00703 | 4988 | 168,986.64 |
| Enalapril \| Propranolol | 0.00803 | 0.00488 | 0.01318 | 47264 | 1,193,480.45 | 0.00803 | 0.00488 | 0.01318 | 15445 | 1,153,109.66 |
| Furosemide \| Hydrochlorothiazide | 0.00172 | 0.00043 | 0.00680 | 10139 | 75,528.64 | 0.00172 | 0.00043 | 0.00680 | 3313 | 50,348.56 |
| Furosemide \| Losartan | 0.02524 | 0.01652 | 0.03837 | 148639 | 2,204,654.19 | 0.02524 | 0.01652 | 0.03837 | 48573 | 1,624,634.38 |
| Furosemide \| Nifedipine | 0.00142 | 0.00038 | 0.00525 | 8358 | 91,242.77 | 0.00142 | 0.00038 | 0.00525 | 2731 | 29,813.83 |
| Furosemide \| Propranolol | 0.00219 | 0.00093 | 0.00518 | 12912 | 327,330.94 | 0.00219 | 0.00093 | 0.00518 | 4219 | 218,110.79 |
| Hydrochlorothiazide \| Losartan | 0.29918 | 0.27134 | 0.32858 | 1761888 | 20,783,471.11 | 0.29918 | 0.27134 | 0.32858 | 575760 | 21,970,478.18 |
| Hydrochlorothiazide \| Nifedipine | 0.01342 | 0.00891 | 0.02017 | 79019 | 622,723.60 | 0.01342 | 0.00891 | 0.02017 | 25822 | 403,562.65 |
| Hydrochlorothiazide \| Propranolol | 0.05533 | 0.04210 | 0.07239 | 325821 | 7,270,621.56 | 0.05533 | 0.04210 | 0.07239 | 106474 | 6,006,101.55 |
| Losartan \| Nifedipine | 0.00634 | 0.00276 | 0.01447 | 37317 | 569,593.12 | 0.00634 | 0.00276 | 0.01447 | 12195 | 413,149.97 |
| Losartan \| Propranolol | 0.01256 | 0.00753 | 0.02087 | 73969 | 2,196,711.19 | 0.01256 | 0.00753 | 0.02087 | 24172 | 1,804,659.55 |
| Nifedipine \| Propranolol | 0.00296 | 0.00108 | 0.00808 | 17441 | 449,668.62 | 0.00296 | 0.00108 | 0.00808 | 5700 | 297,133.23 |
| **Three drugs** |  |  |  |  |  |  |  |  |  |  |
| Amlodipine \| Atenolol \| Captopril | 0.00302 | 0.00089 | 0.01015 | 3756 | 90,668.99 | 0.00302 | 0.00089 | 0.01015 | 1227 | 107,139.13 |
| Amlodipine \| Atenolol \| Enalapril | 0.02318 | 0.01091 | 0.04857 | 28875 | 630,925.31 | 0.02318 | 0.01091 | 0.04857 | 9436 | 776,966.67 |
| Amlodipine \| Atenolol \| Furosemide | 0.00842 | 0.00212 | 0.03284 | 10485 | 230,143.37 | 0.00842 | 0.00212 | 0.03284 | 3426 | 203,431.99 |
| Amlodipine \| Atenolol \| Hydrochlorothiazide | 0.01447 | 0.00578 | 0.03577 | 18022 | 340,861.55 | 0.01447 | 0.00578 | 0.03577 | 5889 | 377,430.03 |
| Amlodipine \| Atenolol \| Losartan | 0.02249 | 0.00696 | 0.07019 | 28009 | 736,541.21 | 0.02249 | 0.00696 | 0.07019 | 9153 | 753,664.26 |
| Amlodipine \| Captopril \| Enalapril | 0.00579 | 0.00083 | 0.03927 | 7210 | 122,132.48 | 0.00579 | 0.00083 | 0.03927 | 2356 | 154,906.46 |
| Amlodipine \| Captopril \| Furosemide | 0.00085 | 0.00012 | 0.00610 | 1054 | 17,958.96 | 0.00085 | 0.00012 | 0.00610 | 345 | 14,761.84 |
| Amlodipine \| Captopril \| Hydrochlorothiazide | 0.02999 | 0.01587 | 0.05597 | 37360 | 523,141.89 | 0.02999 | 0.01587 | 0.05597 | 12209 | 579,924.73 |
| Amlodipine \| Enalapril \| Furosemide | 0.00163 | 0.00023 | 0.01163 | 2026 | 29,882.12 | 0.00163 | 0.00023 | 0.01163 | 662 | 25,030.67 |
| Amlodipine \| Enalapril \| Hydrochlorothiazide | 0.04536 | 0.02646 | 0.07667 | 56491 | 661,689.35 | 0.04536 | 0.02646 | 0.07667 | 18461 | 785,007.87 |
| Amlodipine \| Enalapril \| Losartan | 0.00297 | 0.00041 | 0.02090 | 3695 | 70,560.22 | 0.00297 | 0.00041 | 0.02090 | 1208 | 73,413.18 |
| Amlodipine \| Enalapril \| Propranolol | 0.00490 | 0.00118 | 0.02006 | 6105 | 180,798.19 | 0.00490 | 0.00118 | 0.02006 | 1995 | 157,649.89 |
| Amlodipine \| Furosemide \| Losartan | 0.02698 | 0.01221 | 0.05855 | 33600 | 644,974.91 | 0.02698 | 0.01221 | 0.05855 | 10980 | 415,161.29 |
| Amlodipine \| Furosemide \| Propranolol | 0.00390 | 0.00054 | 0.02761 | 4853 | 144,203.59 | 0.00390 | 0.00054 | 0.02761 | 1586 | 88,912.24 |
| Amlodipine \| Hydrochlorothiazide \| Losartan | 0.12741 | 0.09303 | 0.17210 | 158699 | 2,564,503.70 | 0.12741 | 0.09303 | 0.17210 | 51861 | 2,205,259.37 |
| Amlodipine \| Hydrochlorothiazide \| Propranolol | 0.00543 | 0.00209 | 0.01405 | 6768 | 180,557.93 | 0.00543 | 0.00209 | 0.01405 | 2212 | 134,428.77 |
| Atenolol \| Captopril \| Furosemide | 0.00248 | 0.00057 | 0.01072 | 3085 | 77,183.90 | 0.00248 | 0.00057 | 0.01072 | 1008 | 88,902.85 |
| Atenolol \| Captopril \| Hydrochlorothiazide | 0.02691 | 0.01365 | 0.05237 | 33518 | 736,824.67 | 0.02691 | 0.01365 | 0.05237 | 10953 | 1,017,633.27 |
| Atenolol \| Captopril \| Losartan | 0.00363 | 0.00051 | 0.02548 | 4527 | 132,939.47 | 0.00363 | 0.00051 | 0.02548 | 1479 | 164,404.30 |
| Atenolol \| Enalapril \| Furosemide | 0.01784 | 0.00503 | 0.06122 | 22215 | 504,936.85 | 0.01784 | 0.00503 | 0.06122 | 7260 | 604,177.20 |
| Atenolol \| Enalapril \| Hydrochlorothiazide | 0.11512 | 0.08208 | 0.15916 | 143392 | 2,823,877.32 | 0.11512 | 0.08208 | 0.15916 | 46858 | 4,120,309.14 |
| Atenolol \| Enalapril \| Losartan | 0.00265 | 0.00065 | 0.01074 | 3305 | 89,487.38 | 0.00265 | 0.00065 | 0.01074 | 1080 | 114,676.36 |
| Atenolol \| Furosemide \| Losartan | 0.00732 | 0.00203 | 0.02605 | 9122 | 247,898.64 | 0.00732 | 0.00203 | 0.02605 | 2981 | 248,078.82 |
| Atenolol \| Furosemide \| Nifedipine | 0.00072 | 0.00010 | 0.00517 | 894 | 20,794.85 | 0.00072 | 0.00010 | 0.00517 | 292 | 17,721.35 |
| Atenolol \| Hydrochlorothiazide \| Losartan | 0.17048 | 0.12799 | 0.22347 | 212342 | 5,125,887.62 | 0.17048 | 0.12799 | 0.22347 | 69390 | 6,101,588.86 |
| Atenolol \| Hydrochlorothiazide \| Nifedipine | 0.00917 | 0.00295 | 0.02816 | 11419 | 230,941.49 | 0.00917 | 0.00295 | 0.02816 | 3732 | 244,077.89 |
| Atenolol \| Losartan \| Nifedipine | 0.00401 | 0.00055 | 0.02837 | 4989 | 137,732.68 | 0.00401 | 0.00055 | 0.02837 | 1630 | 136,351.72 |
| Captopril \| Enalapril \| Hydrochlorothiazide | 0.00554 | 0.00134 | 0.02268 | 6899 | 101,984.47 | 0.00554 | 0.00134 | 0.02268 | 2255 | 160,873.75 |
| Captopril \| Furosemide \| Losartan | 0.01100 | 0.00203 | 0.05722 | 13695 | 304,919.18 | 0.01100 | 0.00203 | 0.05722 | 4475 | 298,165.18 |
| Captopril \| Furosemide \| Propranolol | 0.00908 | 0.00269 | 0.03019 | 11311 | 370,815.71 | 0.00908 | 0.00269 | 0.03019 | 3696 | 313,713.12 |
| Captopril \| Hydrochlorothiazide \| Losartan | 0.01614 | 0.00763 | 0.03385 | 20108 | 386,653.99 | 0.01614 | 0.00763 | 0.03385 | 6571 | 468,781.11 |
| Captopril \| Hydrochlorothiazide \| Nifedipine | 0.02064 | 0.00888 | 0.04722 | 25708 | 393,677.12 | 0.02064 | 0.00888 | 0.04722 | 8401 | 410,056.63 |
| Captopril \| Hydrochlorothiazide \| Propranolol | 0.07398 | 0.03474 | 0.15062 | 92142 | 2,740,994.15 | 0.07398 | 0.03474 | 0.15062 | 30111 | 2,697,671.86 |
| Captopril \| Nifedipine \| Propranolol | 0.00413 | 0.00058 | 0.02889 | 5144 | 170,857.96 | 0.00413 | 0.00058 | 0.02889 | 1681 | 143,406.87 |
| Enalapril \| Furosemide \| Hydrochlorothiazide | 0.00280 | 0.00068 | 0.01154 | 3492 | 43,973.01 | 0.00280 | 0.00068 | 0.01154 | 1141 | 49,521.47 |
| Enalapril \| Furosemide \| Losartan | 0.00147 | 0.00020 | 0.01054 | 1832 | 36,595.03 | 0.00147 | 0.00020 | 0.01054 | 599 | 36,929.44 |
| Enalapril \| Furosemide \| Propranolol | 0.00255 | 0.00036 | 0.01809 | 3178 | 96,910.22 | 0.00255 | 0.00036 | 0.01809 | 1038 | 82,938.09 |
| Enalapril \| Hydrochlorothiazide \| Losartan | 0.01774 | 0.00795 | 0.03912 | 22098 | 374,325.05 | 0.01774 | 0.00795 | 0.03912 | 7221 | 479,211.82 |
| Enalapril \| Hydrochlorothiazide \| Nifedipine | 0.01579 | 0.00354 | 0.06746 | 19662 | 256,075.21 | 0.01579 | 0.00354 | 0.06746 | 6425 | 281,628.19 |
| Enalapril \| Hydrochlorothiazide \| Propranolol | 0.02903 | 0.01465 | 0.05671 | 36163 | 992,962.01 | 0.02903 | 0.01465 | 0.05671 | 11818 | 999,963.95 |
| Enalapril \| Nifedipine \| Propranolol | 0.00587 | 0.00105 | 0.03209 | 7310 | 226,065.07 | 0.00587 | 0.00105 | 0.03209 | 2389 | 191,915.97 |
| Furosemide \| Hydrochlorothiazide \| Losartan | 0.00528 | 0.00224 | 0.01236 | 6571 | 111,962.37 | 0.00528 | 0.00224 | 0.01236 | 2147 | 93,183.70 |
| Furosemide \| Hydrochlorothiazide \| Nifedipine | 0.00152 | 0.00021 | 0.01089 | 1891 | 24,816.37 | 0.00152 | 0.00021 | 0.01089 | 618 | 12,898.50 |
| Furosemide \| Losartan \| Nifedipine | 0.00522 | 0.00076 | 0.03510 | 6505 | 133,393.90 | 0.00522 | 0.00076 | 0.03510 | 2126 | 83,172.02 |
| Hydrochlorothiazide \| Losartan \| Nifedipine | 0.02036 | 0.00857 | 0.04759 | 25358 | 443,010.02 | 0.02036 | 0.00857 | 0.04759 | 8287 | 363,245.58 |
| Hydrochlorothiazide \| Losartan \| Propranolol | 0.06048 | 0.03891 | 0.09285 | 75327 | 2,403,256.58 | 0.06048 | 0.03891 | 0.09285 | 24616 | 2,082,849.27 |
| Hydrochlorothiazide \| Nifedipine \| Propranolol | 0.00362 | 0.00069 | 0.01872 | 4509 | 126,201.79 | 0.00362 | 0.00069 | 0.01872 | 1474 | 91,510.61 |
| Losartan \| Nifedipine \| Propranolol | 0.00065 | 0.00009 | 0.00473 | 814 | 28,792.66 | 0.00065 | 0.00009 | 0.00473 | 266 | 21,368.63 |
| **TOTAL** |  |  |  |  | **169,689,247.42** |  |  |  |  | **194,199,292.72** |
