# Supplementary material for: Direct cost of systemic arterial hypertension and its complications in the circulatory system from the perspective of the Brazilian public health system in 2019
Source: PLoS One. 2021 Jun 10;16(6):e0253063. doi: 10.1371/journal.pone.0253063 (PMC8191920; doi:10.1371/journal.pone.0253063)
Supplement: S5 Table — (DOCX) [file pone.0253063.s006.docx]

**S5 Table. Search strategies in scientific databases.**

| Database | Search strategy | Results |
| --- | --- | --- |
| Medline (Pubmed) – 19/04/2020 | ((((((((("Hypertension"[Mesh]) OR Hypertension[Title/Abstract]) OR Blood Pressure, High[Title/Abstract]) OR Blood Pressures, High[Title/Abstract]) OR High Blood Pressure[Title/Abstract]) OR High Blood Pressures[Title/Abstract]) OR Elevated systolic blood[Title/Abstract])) AND (((((((((((((((((((((((((((((("Myocardial Infarction"[Mesh]) OR Myocardial Infarction[Title/Abstract]) OR Infarction, Myocardial[Title/Abstract]) OR Infarctions, Myocardial[Title/Abstract]) OR Myocardial Infarctions[Title/Abstract]) OR Cardiovascular Stroke[Title/Abstract]) OR Cardiovascular Strokes[Title/Abstract]) OR Stroke, Cardiovascular[Title/Abstract]) OR Strokes, Cardiovascular[Title/Abstract]) OR Heart Attack[Title/Abstract]) OR Heart Attacks[Title/Abstract]) OR Myocardial Infarct[Title/Abstract]) OR Infarct, Myocardial[Title/Abstract]) OR Infarcts, Myocardial[Title/Abstract]) OR Myocardial Infarcts[Title/Abstract])) OR (((((((((((((((((((((((((((((("Stroke"[Mesh]) OR Stroke[Title/Abstract]) OR Strokes[Title/Abstract]) OR Cerebrovascular Accident[Title/Abstract]) OR Cerebrovascular Accidents[Title/Abstract]) OR CVA (Cerebrovascular Accident)[Title/Abstract]) OR CVAs (Cerebrovascular Accident)[Title/Abstract]) OR Cerebrovascular Apoplexy[Title/Abstract]) OR Apoplexy, Cerebrovascular[Title/Abstract]) OR Vascular Accident, Brain[Title/Abstract]) OR Brain Vascular Accident[Title/Abstract]) OR Brain Vascular Accidents[Title/Abstract]) OR Vascular Accidents, Brain[Title/Abstract]) OR Cerebrovascular Stroke[Title/Abstract]) OR Cerebrovascular Strokes[Title/Abstract]) OR Stroke, Cerebrovascular[Title/Abstract]) OR Strokes, Cerebrovascular[Title/Abstract]) OR Apoplexy[Title/Abstract]) OR Cerebral Stroke[Title/Abstract]) OR Cerebral Strokes[Title/Abstract]) OR Stroke, Cerebral[Title/Abstract]) OR Strokes, Cerebral[Title/Abstract]) OR Stroke, Acute[Title/Abstract]) OR Acute Stroke[Title/Abstract]) OR Acute Strokes[Title/Abstract]) OR Strokes, Acute[Title/Abstract]) OR Cerebrovascular Accident, Acute[Title/Abstract]) OR Acute Cerebrovascular Accident[Title/Abstract]) OR Acute Cerebrovascular Accidents[Title/Abstract]) OR Cerebrovascular Accidents, Acute[Title/Abstract])) OR ((((("Cardiovascular Diseases"[Mesh]) OR Cardiovascular Diseases[Title/Abstract]) OR Cardiovascular Disease[Title/Abstract]) OR Disease, Cardiovascular[Title/Abstract]) OR Diseases, Cardiovascular[Title/Abstract])) OR (((((((((((("Rheumatic Heart Disease"[Mesh]) OR Rheumatic Heart Disease[Title/Abstract]) OR Disease, Rheumatic Heart[Title/Abstract]) OR Diseases, Rheumatic Heart[Title/Abstract]) OR Heart Disease, Rheumatic[Title/Abstract]) OR Heart Diseases, Rheumatic[Title/Abstract]) OR Rheumatic Heart Diseases[Title/Abstract]) OR Bouillaud Disease[Title/Abstract]) OR Disease, Bouillaud[Title/Abstract]) OR Bouillaud's Disease[Title/Abstract]) OR Bouillauds Disease[Title/Abstract]) OR Disease, Bouillaud's[Title/Abstract])) OR ((((((((((("Myocardial Ischemia"[Mesh]) OR Myocardial Ischemia[Title/Abstract]) OR Ischemia, Myocardial[Title/Abstract]) OR Ischemias, Myocardial[Title/Abstract]) OR Myocardial Ischemias[Title/Abstract]) OR Ischemic Heart Disease[Title/Abstract]) OR Heart Disease, Ischemic[Title/Abstract]) OR Disease, Ischemic Heart[Title/Abstract]) OR Diseases, Ischemic Heart[Title/Abstract]) OR Heart Diseases, Ischemic[Title/Abstract]) OR Ischemic Heart Diseases[Title/Abstract])) OR ((((((((((((((((((((((((((((("Cardiomyopathies"[Mesh]) OR Cardiomyopathies[Title/Abstract]) OR Cardiomyopathy[Title/Abstract]) OR Myocardial Diseases[Title/Abstract]) OR Disease, Myocardial[Title/Abstract]) OR Diseases, Myocardial[Title/Abstract]) OR Myocardial Disease[Title/Abstract]) OR Myocardiopathies[Title/Abstract]) OR Myocardiopathy[Title/Abstract]) OR Cardiomyopathies, Secondary[Title/Abstract]) OR Cardiomyopathy, Secondary[Title/Abstract]) OR Secondary Cardiomyopathies[Title/Abstract]) OR Secondary Cardiomyopathy[Title/Abstract]) OR Secondary Myocardial Diseases[Title/Abstract]) OR Disease, Secondary Myocardial[Title/Abstract]) OR Diseases, Secondary Myocardial[Title/Abstract]) OR Myocardial Disease, Secondary[Title/Abstract]) OR Secondary Myocardial Disease[Title/Abstract]) OR Myocardial Diseases, Secondary[Title/Abstract]) OR Cardiomyopathies, Primary[Title/Abstract]) OR Cardiomyopathy, Primary[Title/Abstract]) OR Primary Cardiomyopathies[Title/Abstract]) OR Primary Cardiomyopathy[Title/Abstract]) OR Primary Myocardial Diseases[Title/Abstract]) OR Myocardial Diseases, Primary[Title/Abstract]) OR Disease, Primary Myocardial[Title/Abstract]) OR Diseases, Primary Myocardial[Title/Abstract]) OR Myocardial Disease, Primary[Title/Abstract]) OR Primary Myocardial Disease[Title/Abstract])) OR (((("Myocarditis"[Mesh]) OR Myocarditis[Title/Abstract]) OR Myocarditides[Title/Abstract]) OR Carditis[Title/Abstract])) OR (("Atrial Fibrillation"[Mesh]) OR (Atrial Fibrillation[Title/Abstract] OR Atrial Fibrillations[Title/Abstract] OR Fibrillation, Atrial[Title/Abstract] OR Fibrillations, Atrial[Title/Abstract] OR Auricular Fibrillation[Title/Abstract] OR Auricular Fibrillations[Title/Abstract] OR Fibrillation, Auricular[Title/Abstract] OR Fibrillations, Auricular[Title/Abstract] OR Persistent Atrial Fibrillation[Title/Abstract] OR Atrial Fibrillation, Persistent[Title/Abstract] OR Atrial Fibrillations, Persistent[Title/Abstract] OR Fibrillation, Persistent Atrial[Title/Abstract] OR Fibrillations, Persistent Atrial[Title/Abstract] OR Persistent Atrial Fibrillations[Title/Abstract] OR Familial Atrial Fibrillation[Title/Abstract] OR Atrial Fibrillation, Familial[Title/Abstract] OR Atrial Fibrillations, Familial[Title/Abstract] OR Familial Atrial Fibrillations[Title/Abstract] OR Fibrillation, Familial Atrial[Title/Abstract] OR Fibrillations, Familial Atrial[Title/Abstract] OR Paroxysmal Atrial Fibrillation[Title/Abstract] OR Atrial Fibrillation, Paroxysmal[Title/Abstract] OR Atrial Fibrillations, Paroxysmal[Title/Abstract] OR Fibrillation, Paroxysmal Atrial[Title/Abstract] OR Fibrillations, Paroxysmal Atrial[Title/Abstract] OR Paroxysmal Atrial Fibrillations[Title/Abstract]))) OR (("Atrial Flutter"[Mesh]) OR (Atrial Flutter[Title/Abstract] OR Atrial Flutters[Title/Abstract] OR Flutter, Atrial[Title/Abstract] OR Flutters, Atrial[Title/Abstract] OR Auricular Flutter[Title/Abstract] OR Auricular Flutters[Title/Abstract] OR Flutter, Auricular[Title/Abstract] OR Flutters, Auricular[Title/Abstract] OR Ventricular Flutter[Title/Abstract] OR Ventricular Flutters[Title/Abstract]))) OR (("Aortic Aneurysm"[Mesh]) OR (Aortic Aneurysm[Title/Abstract] OR Aneurysms, Aortic[Title/Abstract] OR Aortic Aneurysms[Title/Abstract] OR Aneurysm, Aortic[Title/Abstract]))) OR (("Peripheral Vascular Diseases"[Mesh]) OR (Peripheral Vascular Diseases[Title/Abstract] OR Disease, Peripheral Vascular[Title/Abstract] OR Peripheral Vascular Disease[Title/Abstract] OR Vascular Disease, Peripheral[Title/Abstract] OR Peripheral Angiopathies[Title/Abstract] OR Angiopathies, Peripheral[Title/Abstract] OR Angiopathy, Peripheral[Title/Abstract] OR Peripheral Angiopathy[Title/Abstract] OR Vascular Diseases, Peripheral[Title/Abstract] OR Diseases, Peripheral Vascular[Title/Abstract]))) OR (("Endocarditis"[Mesh]) OR (Endocarditis[Title/Abstract] OR Endocarditides[Title/Abstract] OR Infective Endocarditis[Title/Abstract] OR Endocarditides, Infective[Title/Abstract] OR Endocarditis, Infective[Title/Abstract] OR Infective Endocarditides[Title/Abstract]))) OR (("Renal Insufficiency, Chronic"[Mesh]) OR (Renal Insufficiency, Chronic[Title/Abstract] OR Chronic Renal Insufficiencies[Title/Abstract] OR Renal Insufficiencies, Chronic[Title/Abstract] OR Chronic Renal Insufficiency[Title/Abstract] OR Kidney Insufficiency, Chronic[Title/Abstract] OR Chronic Kidney Insufficiency[Title/Abstract] OR Chronic Kidney Insufficiencies[Title/Abstract] OR Kidney Insufficiencies, Chronic[Title/Abstract] OR Chronic Kidney Diseases[Title/Abstract] OR Chronic Kidney Disease[Title/Abstract] OR Disease, Chronic Kidney[Title/Abstract] OR Diseases, Chronic Kidney[Title/Abstract] OR Kidney Disease, Chronic[Title/Abstract] OR Kidney Diseases, Chronic[Title/Abstract] OR Chronic Renal Diseases[Title/Abstract] OR Chronic Renal Disease[Title/Abstract] OR Disease, Chronic Renal[Title/Abstract] OR Diseases, Chronic Renal[Title/Abstract] OR Renal Disease, Chronic[Title/Abstract] OR Renal Diseases, Chronic[Title/Abstract]))) OR Hypertensive heart disease[Title/Abstract]) OR Circulatory diseases[Title/Abstract])) AND (((((((systematic review[ti] OR systematic literature review[ti] OR systematic scoping review[ti] OR systematic narrative review[ti] OR systematic qualitative review[ti] OR systematic evidence review[ti] OR systematic quantitative review[ti] OR systematic meta-review[ti] OR systematic critical review[ti] OR systematic mixed studies review[ti] OR systematic mapping review[ti] OR systematic cochrane review[ti] OR systematic search and review[ti] OR systematic integrative review[ti]) NOT comment[pt] NOT (protocol[ti] OR protocols[ti])) NOT MEDLINE [subset]) OR (Cochrane Database Syst Rev[ta] AND review[pt]) OR systematic review[pt]))) OR (("Meta-Analysis as Topic"[Mesh]) OR (Meta-Analysis as Topic[Title/Abstract] OR Meta Analysis as Topic[Title/Abstract] OR Data Pooling[Title/Abstract] OR Data Poolings[Title/Abstract] OR meta-analyses[Title/Abstract] OR meta-analysis[Title/Abstract] OR cohort pooling[Title/Abstract] OR pooled analysis[Title/Abstract]))) | 5,229 |
| Embase – 19/04/2020 | #1 'hypertension'/exp OR 'htn (hypertension)' OR 'acute hypertension' OR 'arterial hypertension' OR 'blood pressure, high' OR 'cardiovascular hypertension' OR 'controlled hypertension' OR 'endocrine hypertension' OR 'high blood pressure' OR 'high renin hypertension' OR 'hypertension' OR 'hypertensive disease' OR 'hypertensive effect' OR 'hypertensive response' OR 'neurogenic hypertension' OR 'preexistent hypertension' OR 'refractory hypertension' OR 'salt high blood pressure' OR 'salt hypertension' OR 'secondary hypertension' OR 'systemic hypertension'  #2 'heart infarction'/exp OR 'cardiac infarct' OR 'cardiac infarction' OR 'cardial infarct' OR 'heart attack' OR 'heart infarct' OR 'heart infarction' OR 'heart micro infarction' OR 'heart muscle infarction' OR 'infarction, heart' OR 'myocardial infarct' OR 'myocardial infarction' OR 'myocardium infarct' OR 'myocardium infarction' OR 'premonitory infarction sign' OR 'second heart attack' OR 'subendocardial infarction' OR 'transmural cardiac infarction' OR 'transmural heart infarction' OR 'transmural infarction, heart' OR 'cerebrovascular accident'/exp OR 'cva' OR 'accident, cerebrovascular' OR 'acute cerebrovascular lesion' OR 'acute focal cerebral vasculopathy' OR 'acute stroke' OR 'apoplectic stroke' OR 'apoplexia' OR 'apoplexy' OR 'blood flow disturbance, brain' OR 'brain accident' OR 'brain attack' OR 'brain blood flow disturbance' OR 'brain insult' OR 'brain insultus' OR 'brain ischaemic attack' OR 'brain ischemic attack' OR 'brain vascular accident' OR 'cerebral apoplexia' OR 'cerebral insult' OR 'cerebral stroke' OR 'cerebral vascular accident' OR 'cerebral vascular insufficiency' OR 'cerebro vascular accident' OR 'cerebrovascular accident' OR 'cerebrovascular arrest' OR 'cerebrovascular failure' OR 'cerebrovascular injury' OR 'cerebrovascular insufficiency' OR 'cerebrovascular insult' OR 'cerebrum vascular accident' OR 'cryptogenic stroke' OR 'ischaemic cerebral attack' OR 'ischaemic seizure' OR 'ischemic cerebral attack' OR 'ischemic seizure' OR 'stroke' OR 'cardiovascular disease'/exp OR 'angiocardiopathy' OR 'angiocardiovascular disease' OR 'cardiovascular complication' OR 'cardiovascular disease' OR 'cardiovascular diseases' OR 'cardiovascular disorder' OR 'cardiovascular disturbance' OR 'cardiovascular lesion' OR 'cardiovascular syndrome' OR 'cardiovascular vegetative disorder' OR 'complication, cardiovascular' OR 'disease, cardiovascular' OR 'major adverse cardiovascular event' OR 'rheumatic heart disease'/exp OR 'heart disease, rheumatic' OR 'rheumatic cardiac disease' OR 'rheumatic cardiopathy' OR 'rheumatic heart disease' OR 'rheumatic valve disease' OR 'rheumatic valvular disease' OR 'rheumatoid heart disease' OR 'heart muscle ischemia'/exp OR 'acute heart muscle ischaemia' OR 'acute heart muscle ischemia' OR 'cardiac ischaemia' OR 'cardiac ischemia' OR 'cardiac muscle ischaemia' OR 'cardiac muscle ischemia' OR 'coronary artery ischaemia' OR 'coronary artery ischemia' OR 'coronary ischaemia' OR 'coronary ischemia' OR 'coronary syndrome' OR 'heart anoxia' OR 'heart hypoxia' OR 'heart ischaemia' OR 'heart ischaemic arrest' OR 'heart ischaemic attack' OR 'heart ischaemic time' OR 'heart ischemia' OR 'heart ischemic arrest' OR 'heart ischemic attack' OR 'heart ischemic time' OR 'heart muscle hypoxia' OR 'heart muscle ischaemia' OR 'heart muscle ischaemia, subepicardial' OR 'heart muscle ischemia' OR 'heart muscle ischemia, subepicardial' OR 'heart transient ischaemic attack' OR 'heart transient ischemic attack' OR 'hypoxia, heart' OR 'hypoxic heart' OR 'ischaemic heart' OR 'ischaemic heart arrest' OR 'ischaemic myocardium' OR 'ischaemic time' OR 'ischemic heart' OR 'ischemic heart arrest' OR 'ischemic myocardium' OR 'ischemic time' OR 'myocardial anoxia' OR 'myocardial hypoxia' OR 'myocardial ischaemia' OR 'myocardial ischemia' OR 'myocardium hypoxia' OR 'myocardium ischaemia' OR 'myocardium ischemia' OR 'subendocardial ischaemia' OR 'subendocardial ischemia' OR 'transient ischaemic attack, heart' OR 'transient ischemic attack, heart' OR 'cardiomyopathy'/exp OR 'cardiomyopathies' OR 'cardiomyopathy' OR 'heart myopathy' OR 'myocardiopathy' OR 'primary myocardial disease' OR 'myocarditis'/exp OR 'allergic myocarditis' OR 'inflammation, myocardial' OR 'myocard inflammation' OR 'myocardial inflammation' OR 'myocarditis' OR 'atrial fibrillation'/exp OR 'atrial fibrillation' OR 'atrium fibrillation' OR 'auricular fibrilation' OR 'auricular fibrillation' OR 'cardiac atrial fibrillation' OR 'cardiac atrium fibrillation' OR 'fibrillation, heart atrium' OR 'heart atrial fibrillation' OR 'heart atrium fibrillation' OR 'heart fibrillation atrium' OR 'non-valvular atrial fibrillation' OR 'nonvalvular atrial fibrillation' OR 'heart atrium flutter'/exp OR 'atrial flutter' OR 'atrium flutter' OR 'atrium flutter, heart' OR 'auricular flutter' OR 'cardiac atrial flutter' OR 'cardiac atrium flutter' OR 'flutter, heart atrium' OR 'heart atrial flutter' OR 'heart atrium flutter' OR 'supraventricular flutter' OR 'aortic aneurysm'/exp OR 'aneurysm, aorta' OR 'aneurysma aortae' OR 'aorta aneurysm' OR 'aorta aneurysm resection' OR 'aortic aneurysm' OR 'peripheral vascular disease'/exp OR 'peripheral arteriopathy' OR 'peripheral blood vessel disease' OR 'peripheral vascular disease' OR 'peripheral vascular diseases' OR 'peripheral vascular disorder' OR 'peripheral vasculopathy' OR 'peripheral vessel disease' OR 'endocarditis'/exp OR 'endocardial inflammation' OR 'endocarditis' OR 'paraneoplastic endocarditis' OR 'parietal fibroplastic endocarditis' OR 'chronic kidney failure'/exp OR 'chronic kidney disease' OR 'chronic kidney disorder' OR 'chronic kidney failure' OR 'chronic kidney insufficiency' OR 'chronic nephropathy' OR 'chronic renal disease' OR 'chronic renal failure' OR 'chronic renal insufficiency' OR 'kidney chronic failure' OR 'kidney disease, chronic' OR 'kidney failure, chronic' OR 'kidney function, chronic disease' OR 'renal insufficiency, chronic' OR 'hypertensive heart disease'/exp OR 'circulatory diseases'  #3 'systematic review'/exp OR 'review, systematic' OR 'systematic review' OR 'meta analysis'/exp OR 'analysis, meta' OR 'meta analysis' OR 'meta-analysis' OR 'metaanalysis' OR 'data pooling' OR 'data poolings' OR 'cohort pooling' OR 'pooled analysis'/exp  #4 #1 AND #2 AND #3 | 7,326 |
| Cochrane Library – 19/04/2020 | #1 MeSH descriptor: [Hypertension] explode all trees  #2 (Hypertension):ti,ab,kw (Word variations have been searched)  #3 (Blood Pressure, High):ti,ab,kw (Word variations have been searched)  #4 (High Blood Pressure):ti,ab,kw (Word variations have been searched)  #5 (High Blood Pressures):ti,ab,kw (Word variations have been searched)  #6 (Blood Pressures, High):ti,ab,kw (Word variations have been searched)  #7 (Elevated systolic blood):ti,ab,kw (Word variations have been searched)  #8 {OR #1-#7}  #9 MeSH descriptor: [Myocardial Infarction] explode all trees  #10 (Myocardial Infarction):ti,ab,kw (Word variations have been searched)  #11 (Strokes, Cardiovascular):ti,ab,kw (Word variations have been searched)  #12 (Myocardial Infarct):ti,ab,kw (Word variations have been searched)  #13 (Cardiovascular Stroke):ti,ab,kw (Word variations have been searched)  #14 (Infarcts, Myocardial):ti,ab,kw (Word variations have been searched)  #15 (Infarctions, Myocardial):ti,ab,kw (Word variations have been searched)  #16 (Stroke, Cardiovascular):ti,ab,kw (Word variations have been searched)  #17 (Myocardial Infarcts):ti,ab,kw (Word variations have been searched)  #18 (Heart Attack):ti,ab,kw (Word variations have been searched)  #19 (Myocardial Infarctions):ti,ab,kw (Word variations have been searched)  #20 (Infarct, Myocardial):ti,ab,kw (Word variations have been searched)  #21 (Infarction, Myocardial):ti,ab,kw (Word variations have been searched)  #22 (Heart Attacks):ti,ab,kw (Word variations have been searched)  #23 (Cardiovascular Strokes):ti,ab,kw (Word variations have been searched)  #24 MeSH descriptor: [Stroke] explode all trees  #25 (Stroke):ti,ab,kw (Word variations have been searched)  #26 (Acute Stroke):ti,ab,kw (Word variations have been searched)  #27 (Acute Cerebrovascular Accidents):ti,ab,kw (Word variations have been searched)  #28 (Cerebrovascular Accident, Acute):ti,ab,kw (Word variations have been searched)  #29 (Cerebrovascular Accidents, Acute):ti,ab,kw (Word variations have been searched)  #30 (Acute Strokes):ti,ab,kw (Word variations have been searched)  #31 (Stroke, Acute):ti,ab,kw (Word variations have been searched)  #32 (Acute Cerebrovascular Accident):ti,ab,kw (Word variations have been searched)  #33 (Strokes, Acute):ti,ab,kw (Word variations have been searched)  #34 (Cerebrovascular Stroke):ti,ab,kw (Word variations have been searched)  #35 (Strokes, Cerebrovascular):ti,ab,kw (Word variations have been searched)  #36 (Brain Vascular Accidents):ti,ab,kw (Word variations have been searched)  #37 (CVA (Cerebrovascular Accident)):ti,ab,kw (Word variations have been searched)  #38 (Cerebrovascular Apoplexy):ti,ab,kw (Word variations have been searched)  #39 (Cerebrovascular Accident):ti,ab,kw (Word variations have been searched)  #40 (Vascular Accidents, Brain):ti,ab,kw (Word variations have been searched)  #41 (Cerebrovascular Accidents):ti,ab,kw (Word variations have been searched)  #42 (Brain Vascular Accident):ti,ab,kw (Word variations have been searched)  #43 (Cerebral Stroke):ti,ab,kw (Word variations have been searched)  #44 (Cerebrovascular Strokes):ti,ab,kw (Word variations have been searched)  #45 (Cerebral Strokes):ti,ab,kw (Word variations have been searched)  #46 (Stroke, Cerebral):ti,ab,kw (Word variations have been searched)  #47 (CVAs (Cerebrovascular Accident)):ti,ab,kw (Word variations have been searched)  #48 (Strokes):ti,ab,kw (Word variations have been searched)  #49 (Vascular Accident, Brain):ti,ab,kw (Word variations have been searched)  #50 (Apoplexy):ti,ab,kw (Word variations have been searched)  #51 (Stroke, Cerebrovascular):ti,ab,kw (Word variations have been searched)  #52 (Strokes, Cerebral):ti,ab,kw (Word variations have been searched)  #53 (Apoplexy, Cerebrovascular):ti,ab,kw (Word variations have been searched)  #54 MeSH descriptor: [Cardiovascular Diseases] explode all trees  #55 (Cardiovascular Diseases):ti,ab,kw (Word variations have been searched)  #56 (Diseases, Cardiovascular):ti,ab,kw (Word variations have been searched)  #57 (Cardiovascular Disease):ti,ab,kw (Word variations have been searched)  #58 (Disease, Cardiovascular):ti,ab,kw (Word variations have been searched)  #59 MeSH descriptor: [Rheumatic Heart Disease] explode all trees  #60 (Rheumatic Heart Disease):ti,ab,kw (Word variations have been searched)  #61 (Disease, Rheumatic Heart):ti,ab,kw (Word variations have been searched)  #62 (Heart Diseases, Rheumatic):ti,ab,kw (Word variations have been searched)  #63 (Rheumatic Heart Diseases):ti,ab,kw (Word variations have been searched)  #64 (Heart Disease, Rheumatic):ti,ab,kw (Word variations have been searched)  #65 (Diseases, Rheumatic Heart):ti,ab,kw (Word variations have been searched)  #66 (Disease, Bouillaud):ti,ab,kw (Word variations have been searched)  #67 (Disease, Bouillaud's):ti,ab,kw (Word variations have been searched)  #68 (Bouillauds Disease):ti,ab,kw (Word variations have been searched)  #69 (Bouillaud's Disease):ti,ab,kw (Word variations have been searched)  #70 (Bouillaud Disease):ti,ab,kw (Word variations have been searched)  #71 MeSH descriptor: [Myocardial Ischemia] explode all trees  #72 (Myocardial Ischemia):ti,ab,kw (Word variations have been searched)  #73 (Ischemia, Myocardial):ti,ab,kw (Word variations have been searched)  #74 (Ischemic Heart Diseases):ti,ab,kw (Word variations have been searched)  #75 (Disease, Ischemic Heart):ti,ab,kw (Word variations have been searched)  #76 (Heart Diseases, Ischemic):ti,ab,kw (Word variations have been searched)  #77 (Ischemias, Myocardial):ti,ab,kw (Word variations have been searched)  #78 (Ischemic Heart Disease):ti,ab,kw (Word variations have been searched)  #79 (Diseases, Ischemic Heart):ti,ab,kw (Word variations have been searched)  #80 (Heart Disease, Ischemic):ti,ab,kw (Word variations have been searched)  #81 (Myocardial Ischemias):ti,ab,kw (Word variations have been searched)  #82 MeSH descriptor: [Cardiomyopathies] explode all trees  #83 (Cardiomyopathies):ti,ab,kw (Word variations have been searched)  #84 (Myocardial Diseases):ti,ab,kw (Word variations have been searched)  #85 (Myocardiopathies):ti,ab,kw (Word variations have been searched)  #86 (Myocardial Disease):ti,ab,kw (Word variations have been searched)  #87 (Disease, Myocardial):ti,ab,kw (Word variations have been searched)  #88 (Cardiomyopathy):ti,ab,kw (Word variations have been searched)  #89 (Myocardiopathy):ti,ab,kw (Word variations have been searched)  #90 (Diseases, Myocardial):ti,ab,kw (Word variations have been searched)  #91 (Primary Myocardial Disease):ti,ab,kw (Word variations have been searched)  #92 (Diseases, Primary Myocardial):ti,ab,kw (Word variations have been searched)  #93 (Myocardial Diseases, Primary):ti,ab,kw (Word variations have been searched)  #94 (Primary Myocardial Diseases):ti,ab,kw (Word variations have been searched)  #95 (Cardiomyopathies, Primary):ti,ab,kw (Word variations have been searched)  #96 (Cardiomyopathy, Primary):ti,ab,kw (Word variations have been searched)  #97 (Disease, Primary Myocardial):ti,ab,kw (Word variations have been searched)  #98 (Myocardial Disease, Primary):ti,ab,kw (Word variations have been searched)  #99 (Primary Cardiomyopathies):ti,ab,kw (Word variations have been searched)  #100 (Primary Cardiomyopathy):ti,ab,kw (Word variations have been searched)  #101 (Cardiomyopathies, Secondary):ti,ab,kw (Word variations have been searched)  #102 (Diseases, Secondary Myocardial):ti,ab,kw (Word variations have been searched)  #103 (Secondary Cardiomyopathy):ti,ab,kw (Word variations have been searched)  #104 (Secondary Myocardial Diseases):ti,ab,kw (Word variations have been searched)  #105 (Myocardial Disease, Secondary):ti,ab,kw (Word variations have been searched)  #106 (Myocardial Diseases, Secondary):ti,ab,kw (Word variations have been searched)  #107 (Disease, Secondary Myocardial):ti,ab,kw (Word variations have been searched)  #108 (Secondary Cardiomyopathies):ti,ab,kw (Word variations have been searched)  #109 (Cardiomyopathy, Secondary):ti,ab,kw (Word variations have been searched)  #110 (Secondary Myocardial Disease):ti,ab,kw (Word variations have been searched)  #111 MeSH descriptor: [Myocarditis] explode all trees  #112 (Myocarditis):ti,ab,kw (Word variations have been searched)  #113 (Myocarditides):ti,ab,kw (Word variations have been searched)  #114 (Carditis):ti,ab,kw (Word variations have been searched)  #115 MeSH descriptor: [Atrial Fibrillation] explode all trees  #116 (Atrial Fibrillation):ti,ab,kw (Word variations have been searched)  #117 (Atrial Fibrillation, Paroxysmal):ti,ab,kw (Word variations have been searched)  #118 (Fibrillation, Paroxysmal Atrial):ti,ab,kw (Word variations have been searched)  #119 (Fibrillations, Paroxysmal Atrial):ti,ab,kw (Word variations have been searched)  #120 (Atrial Fibrillations, Paroxysmal):ti,ab,kw (Word variations have been searched)  #121 (Paroxysmal Atrial Fibrillations):ti,ab,kw (Word variations have been searched)  #122 (Paroxysmal Atrial Fibrillation):ti,ab,kw (Word variations have been searched)  #123 (Familial Atrial Fibrillations):ti,ab,kw (Word variations have been searched)  #124 (Fibrillation, Familial Atrial):ti,ab,kw (Word variations have been searched)  #125 (Atrial Fibrillation, Familial):ti,ab,kw (Word variations have been searched)  #126 (Familial Atrial Fibrillation):ti,ab,kw (Word variations have been searched)  #127 (Fibrillations, Familial Atrial):ti,ab,kw (Word variations have been searched)  #128 (Atrial Fibrillations, Familial):ti,ab,kw (Word variations have been searched)  #129 (Fibrillation, Auricular):ti,ab,kw (Word variations have been searched)  #130 (Fibrillations, Atrial):ti,ab,kw (Word variations have been searched)  #131 (Fibrillations, Auricular):ti,ab,kw (Word variations have been searched)  #132 (Fibrillation, Atrial):ti,ab,kw (Word variations have been searched)  #133 (Auricular Fibrillations):ti,ab,kw (Word variations have been searched)  #134 (Atrial Fibrillations):ti,ab,kw (Word variations have been searched)  #135 (Auricular Fibrillation):ti,ab,kw (Word variations have been searched)  #136 (Atrial Fibrillation, Persistent):ti,ab,kw (Word variations have been searched)  #137 (Fibrillations, Persistent Atrial):ti,ab,kw (Word variations have been searched)  #138 (Persistent Atrial Fibrillations):ti,ab,kw (Word variations have been searched)  #139 (Persistent Atrial Fibrillation):ti,ab,kw (Word variations have been searched)  #140 (Atrial Fibrillations, Persistent):ti,ab,kw (Word variations have been searched)  #141 (Fibrillation, Persistent Atrial):ti,ab,kw (Word variations have been searched)  #142 MeSH descriptor: [Atrial Flutter] explode all trees  #143 (Atrial Flutter):ti,ab,kw (Word variations have been searched)  #144 (Flutter, Auricular):ti,ab,kw (Word variations have been searched)  #145 (Atrial Flutters):ti,ab,kw (Word variations have been searched)  #146 (Flutters, Auricular):ti,ab,kw (Word variations have been searched)  #147 (Flutter, Atrial):ti,ab,kw (Word variations have been searched)  #148 (Auricular Flutter):ti,ab,kw (Word variations have been searched)  #149 (Auricular Flutters):ti,ab,kw (Word variations have been searched)  #150 (Flutters, Atrial):ti,ab,kw (Word variations have been searched)  #151 (Ventricular Flutter):ti,ab,kw (Word variations have been searched)  #152 (Ventricular Flutters):ti,ab,kw (Word variations have been searched)  #153 MeSH descriptor: [Aortic Aneurysm] explode all trees  #154 (Aortic Aneurysm):ti,ab,kw (Word variations have been searched)  #155 (Aortic Aneurysms):ti,ab,kw (Word variations have been searched)  #156 (Aneurysms, Aortic):ti,ab,kw (Word variations have been searched)  #157 (Aneurysm, Aortic):ti,ab,kw (Word variations have been searched)  #158 (Peripheral Vascular Diseases):ti,ab,kw (Word variations have been searched)  #159 (Vascular Diseases, Peripheral):ti,ab,kw (Word variations have been searched)  #160 (Angiopathies, Peripheral):ti,ab,kw (Word variations have been searched)  #161 (Vascular Disease, Peripheral):ti,ab,kw (Word variations have been searched)  #162 (Angiopathy, Peripheral):ti,ab,kw (Word variations have been searched)  #163 (Peripheral Angiopathy):ti,ab,kw (Word variations have been searched)  #164 (Peripheral Angiopathies):ti,ab,kw (Word variations have been searched)  #165 (Peripheral Vascular Disease):ti,ab,kw (Word variations have been searched)  #166 (Diseases, Peripheral Vascular):ti,ab,kw (Word variations have been searched)  #167 (Disease, Peripheral Vascular):ti,ab,kw (Word variations have been searched)  #168 MeSH descriptor: [Endocarditis] explode all trees  #169 (Endocarditis):ti,ab,kw (Word variations have been searched)  #170 (Endocarditis, Infective):ti,ab,kw (Word variations have been searched)  #171 (Infective Endocarditis):ti,ab,kw (Word variations have been searched)  #172 (Infective Endocarditides):ti,ab,kw (Word variations have been searched)  #173 (Endocarditides, Infective):ti,ab,kw (Word variations have been searched)  #174 (Endocarditides):ti,ab,kw (Word variations have been searched)  #175 MeSH descriptor: [Renal Insufficiency, Chronic] explode all trees  #176 (Renal Insufficiency, Chronic):ti,ab,kw (Word variations have been searched)  #177 (Diseases, Chronic Renal):ti,ab,kw (Word variations have been searched)  #178 (Renal Disease, Chronic):ti,ab,kw (Word variations have been searched)  #179 (Renal Diseases, Chronic):ti,ab,kw (Word variations have been searched)  #180 (Chronic Renal Disease):ti,ab,kw (Word variations have been searched)  #181 (Diseases, Chronic Kidney):ti,ab,kw (Word variations have been searched)  #182 (Chronic Kidney Disease):ti,ab,kw (Word variations have been searched)  #183 (Disease, Chronic Kidney):ti,ab,kw (Word variations have been searched)  #184 (Chronic Renal Diseases):ti,ab,kw (Word variations have been searched)  #185 (Kidney Disease, Chronic):ti,ab,kw (Word variations have been searched)  #186 (Disease, Chronic Renal):ti,ab,kw (Word variations have been searched)  #187 (Kidney Diseases, Chronic):ti,ab,kw (Word variations have been searched)  #188 (Chronic Kidney Diseases):ti,ab,kw (Word variations have been searched)  #189 (Chronic Kidney Insufficiencies):ti,ab,kw (Word variations have been searched)  #190 (Kidney Insufficiency, Chronic):ti,ab,kw (Word variations have been searched)  #191 (Chronic Kidney Insufficiency):ti,ab,kw (Word variations have been searched)  #192 (Chronic Renal Insufficiency):ti,ab,kw (Word variations have been searched)  #193 (Chronic Renal Insufficiencies):ti,ab,kw (Word variations have been searched)  #194 (Renal Insufficiencies, Chronic):ti,ab,kw (Word variations have been searched)  #195 (Kidney Insufficiencies, Chronic):ti,ab,kw (Word variations have been searched)  #196 (Hypertensive heart disease):ti,ab,kw (Word variations have been searched)  #197 (Circulatory diseases):ti,ab,kw (Word variations have been searched)  #198 {OR #9-#197}  #199 #8 AND #198  Filter: Cochrane Reviews | 319 |
| TOTAL |  | 12,874 |
