# Supplementary material for: Direct cost of systemic arterial hypertension and its complications in the circulatory system from the perspective of the Brazilian public health system in 2019
Source: PLoS One. 2021 Jun 10;16(6):e0253063. doi: 10.1371/journal.pone.0253063 (PMC8191920; doi:10.1371/journal.pone.0253063)
Supplement: S6 Table — (DOCX) [file pone.0253063.s007.docx]

**S6 Table. Critical appraisal of the systematic review studies using AMSTAR 2.**

|  | Poorzand et al, 2019 | Yang et al, 2015 | Feigin et al, 2005 | Ariesen et al, 2003 | Wang et al, 2017 | Ji et al, 2019 | Kobeissi et al, 2019 | Song et al, 2019 |
| --- | --- | --- | --- | --- | --- | --- | --- | --- |
| 1. Did the research questions and inclusion criteria for the review include the components of PICO? | Yes | Yes | Yes | Yes | Yes | Yes | Yes | Yes |
| 2. Did the report of the review contain an explicit statement that the review methods were established prior to the conduct of the review and did the report justify any significant deviations from the protocol? | Yes | No | No | No | No | No | Yes | No |
| 3. Did the review authors explain their selection of the study designs for inclusion in the review? | Yes | Yes | Yes | Yes | Yes | Yes | Yes | Yes |
| 4. Did the review authors use a comprehensive literature search strategy? | Partial Yes | Partial Yes | No | Partial Yes | Partial Yes | No | Partial Yes | Yes |
| 5. Did the review authors perform study selection in duplicate? | Yes | Yes | Yes | No | Yes | Yes | Yes | No |
| 6. Did the review authors perform data extraction in duplicate? | Yes | Yes | Yes | Yes | Yes | Yes | Yes | No |
| 7. Did the review authors provide a list of excluded studies and justify the exclusions? | No | No | No | No | No | No | No | No |
| 8. Did the review authors describe the included studies in adequate detail? | Yes | Yes | Yes | Partial Yes | Partial Yes | Yes | Yes | Yes |
| 9. Did the review authors use a satisfactory technique for assessing the risk of bias (RoB) in individual studies that were included in the review? | Yes | Partial Yes | No | No | Partial Yes | Partial Yes | Partial Yes | No |
| 10. Did the review authors report on the sources of funding for the studies included in the review? | No | No | No | No | No | No | No | No |
| 11. If meta-analysis was performed did the review authors use appropriate methods for statistical combination of results? | Yes | Yes | Yes | Yes | Yes | Yes | Yes | Yes |
| 12. If meta-analysis was performed, did the review authors assess the potential impact of RoB in individual studies on the results of the meta-analysis or other evidence synthesis? | No | No | No | No | No | No | No | No |
| 13. Did the review authors account for RoB in individual studies when interpreting/ discussing the results of the review? | No | No | No | No | No | Yes | Yes | No |
| 14. Did the review authors provide a satisfactory explanation for, and discussion of, any heterogeneity observed in the results of the review? | Yes | Yes | No | No | Yes | Yes | Yes | No |
| 15. If they performed quantitative synthesis did the review authors carry out an adequate investigation of publication bias (small study bias) and discuss its likely impact on the results of the review? | Yes | Yes | No | No | Yes | Yes | Yes | No |
| 16. Did the review authors report any potential sources of conflict of interest, including any funding they received for conducting the review? | Yes | Yes | Yes | Yes | No | Yes | Yes | Yes |
| **Conclusion** | Low quality | Low quality | Critically Low quality | Critically Low quality | Low quality | Low quality | Moderate quality | Critically Low quality |
