# Supplementary material for: Direct cost of systemic arterial hypertension and its complications in the circulatory system from the perspective of the Brazilian public health system in 2019
Source: PLoS One. 2021 Jun 10;16(6):e0253063. doi: 10.1371/journal.pone.0253063 (PMC8191920; doi:10.1371/journal.pone.0253063)
Supplement: Attachment — Submitted filename: Response to Reviewers.docx [file pone.0253063.s009.docx]

Dear Editor,

We thank you for the opportunity to have our manuscript **PONE-D-20-37639R1**, entitled "Direct cost of systemic arterial hypertension and its complications in the circulatory system from the perspective of the Brazilian public health system in 2019", evaluated for publication by PlOS ONE. After analyzing all the comments made, we have sought to respond to them appropriately, as described below.

Yours sincerely,

The Authors

**Editor, comment 1: “**Please review your reference list to ensure that it is complete and correct. If you have cited papers that have been retracted, please include the rationale for doing so in the manuscript text, or remove these references and replace them with relevant current references. Any changes to the reference list should be mentioned in the rebuttal letter that accompanies your revised manuscript. If you need to cite a retracted article, indicate the article’s retracted status in the References list and also include a citation and full reference for the retraction notice. “

**Our response:** Thank you for the information. Our reference list is complete and correct.

**Reviewer 2, comment 1:** “Dear Authors, congratulations for this important scientific communication. I appreciate your attention to my previous requests. Nevertheless, two minor observations prevail: 1) on page 5, line 99, you state that the SUS provides essential medications, when there are evaluations based on national data that show limitations in coverage of health care (https://www.scielosp.org/article/rsp/2017.v51suppl1/3s/#). 2) On page 21, line 423, you suggest that coverage of primary care is successful for SAH. Again, some sources show that mortality due to this health need is considerable (https://www.scielosp.org/article/rsp/2014.v48n4/671-681/en/#). Perhaps you could suggest a way to achieve a better scenario.”

**Our response:** Thank you for the comments. We made the suggested changes. 1) On page 5, line 105, we added the information about barriers to access to medication in Brazil, as follows:

“Although there is a legal obligation to provide drugs free of charge, the population has faced barriers to access to medication in Brazil, not achieving full coverage (35).”

2) We moderated the statement on the contribution of primary care to SAH management in Brazil, as the reviewer suggested. We removed the word “substantial” from the following sentence:

"Costs related to complications of SAH were relatively low in our study, probably due to the SAH care coverage in primary health care, provided free of charge.”

In regard to the article cited by the reviewer, we added information on improving health information systems to page 23, line 462 of the revised manuscript:

“Finally, it is important to note that there is still a need to improve the health information systems used to manage diseases in Brazil (27). Disparities remain in the health information records of the different Brazilian institutions, in addition to difficulties related to the technological infrastructure and qualification of the professionals involved in data collection (93). Thus, it is important to continuously modernize Brazilian information systems, as well as professional qualification and awareness of the relevance of ensuring that the health registers are complete.”
